# Synergistic Effects of Two-Dimensional MXene and Ammonium Polyphosphate on Enhancing the Fire Safety of Polyvinyl Alcohol Composite Aerogels

**DOI:** 10.3390/polym11121964

**Published:** 2019-11-29

**Authors:** Xinxin Sheng, Sihao Li, Yanfeng Zhao, Dongsheng Zhai, Li Zhang, Xiang Lu

**Affiliations:** 1 Guangdong Provincial Key Laboratory of Functional Soft Condensed Matter, School of Materials and Energy, Guangdong University of Technology, Guangzhou 510006, China; 2111902041@mail2.gdut.edu.cn (S.L.); 2017021930@m.scnu.edu.cn (Y.Z.); dongshengzhai567@126.com (D.Z.); lizhang@gdut.edu.cn (L.Z.); 2 School of Chemistry and Environment, South China Normal University, Guangzhou 510006, China; 3 Key Laboratory of Polymer Processing Engineering of the Ministry of Education, National Engineering Research Center of Novel Equipment for Polymer Processing, South China University of Technology, Guangzhou 510641, China

**Keywords:** MXene, synergistic effect, ammonium polyphosphate, polyvinyl alcohol (PVA)

## Abstract

Fire and smoke suppressions of polyvinyl alcohol (PVA) aerogels are urgently required due to the serious fire hazard they present. MXene, a 2D transition-metal carbide with many excellent properties, is considered a promising synergist for providing excellent flame retardant performance. PVA/ammonium polyphosphate (APP)/transition metal carbide (MXene) composite aerogels were prepared via the freeze-drying method to enhance the flame retardancy. Thermogravimetric analysis, limiting oxygen index, vertical burning, and cone calorimeter tests were executed to investigate the thermal stability and flame retardancy of PVA/APP/MXene (PAM) composite aerogels. The results demonstrated that MXene boosted the flame retardancy of PVA-APP, and that PAM-2 (with 2.0 wt% MXene loading) passed the V-0 rating, and reached a maximum LOI value of 42%; Moreover, MXene endowed the PVA-APP system with excellent fire and smoke suppression performance, as the the peak heat release rate and peak smoke production rate were significantly reduced by 55% and 74% at 1.0 wt% MXene loading. The flame retardant mechanism was systematically studied, MXene facilitated the generation of compact intumescent residues via ita catalyst effects, thus further restraining the release of heat and smoke. This work provides a simple route to improve the flame retardancy of PVA aerogels via the synergistic effect of MXene and APP.

## 1. Introduction

Aerogels are porous solid materials composed of nanoscale colloidal particles or polymer materials [1]. Their unique open nanoscale porous structure and continuous three-dimensional network structure give them significantly low density, high specific surface area and high porosity, wherein the solid phase of the aerogel accounts for only about 0.2–20% of the total volume. Due to the strong adsorption ability, low thermal conductivity, low acoustic impedance and low refractive index, etc., most aerogels have broad application prospects in aviation and aerospace, chemical industry, metallurgical industry, energy-saving buildings, and so on [2,3].

Polyvinyl alcohol (PVA) is a water-soluble and very biocompatible polymer material [4,5], which is often used to prepare organic aerogels by freeze-drying. Compared to inorganic aerogels, PVA organic aerogels usually exhibit better fracture toughness. However, PVA is very easily ignited and has a quite fast fire spread speed owing to its organic structure, and its LOI is only around 20.0%. The flammability of PVA aerogels restricts their effective application in some fields that require high flame retardancy, thus it is very essential to improve the flame retardancy of PVA aerogels.

In general, the incorporation of flame retardants (FR) into polymer materials is the most effective method to lower the flammability of polymer-based materials. Among all FRs, intumescent flame retardants (IFRs), such as ammonium polyphosphate (APP), are considered to be some of the most promising, eco-friendly, and halogen-free flame retardants thanks to the non-toxicity and anti-dripping properties during the combustion process. However, the traditional IFRs have bad flame retardant efficiency and thermal stability. Compared to halogenated flame retardants, higher mass fractions of halogen-free flame retardants are required to achieve the same flame retardant rating for the polymer-based materials, and the use of a great quantity of halogen-free flame retardant not only causes a decrease in the physical properties of the resulting material, but also causes an increase in material cost. In order to solve these problems, synergists are incorporated to enhance the flame retardancy of IFR and reduce the amount of the IFR required in the polymer matrix.

Li et al. [6] prepared a series of zeolites (ZEO) and APP synergistic flame retardant polyurethane foams (PUFs), and investigated the effect of ZEO on the mechanical performance, thermal performance, flame retardant properties of the PUF/APP composites; the results demonstrated that, with the introduction of ZEO, the flame retardancy of PUF/APP composites was obviously improved, and a synergistic effect between ZEO and APP was observed. Liu et al. [7] found that there is obviously synergistic effect between lanthanum oxide (La_2_O_3_) and IFR for polylactide (PLA)/IFR composites, as the flame retardant performance indices, such as limited oxygen index (LOI) and vertical burning (UL-94), of the PLA/IFR composite were dramatically enhanced with the addition of La_2_O_3_. Feng et al. [8] synthesized a series of maleated cyclodextrins (MCs) and their metal salts (metal MCs), and used them as the synergists for PVA/IFR composites. The results show that the metal MCs could facilitate the generation of an organized and dense char layer in the condensed phase during the degradation of PVA, and the thermal stability and vertical burning level of the PVA/IFR composites was greatly improved. All the above studies show that the addition of a small amount of synergist to a polymer/IFR composite is an effective way to significantly improve the flame retardant properties of the polymer/IFR composite.

MXene is a new type of two-dimensional (2D) layered transition metal carbide/carbonitride, where M represents early transition metal and X stands for carbon and/or nitrogen. Ti_3_C_2_T_x_ (T represents the surface termination, including −O, −OH, or −F) is a kind of widely studied MXene, due to its good electromagnetic, electronic conductivity, chemical stability, antibacterial properties [9,10,11]. Sheng et al. [12] proved that the incorporation of exfoliated MXene nanosheets resulted in the significant enhancement on the mechanical and thermal performances of thermoplastic polyurethane (TPU). Wang et al. [9] fabricated an annealed Ti_3_C_2_T_x_ MXene/epoxy nanocomposite, and found that it exhibited an excellent EMI shielding effectiveness (SE) and electrical conductivity. Yu et al. [13] revealed that the addition of cetyltrimethylammonium bromide (CTAB)- and tetrabutylphosphine chloride (TBPC)-functionalized MXene nanomaterials could remarkably enhance the fire safety and smoke suppression properties of TPU. However, the strategy for the fabrication of MXene-based flame retardant polymers has the drawback of complex manipulation, and it is thus difficult to industrialize the production.

To date, there have been no reports on the synergistic flame retardant effect of MXene on the polymer/halogen-free flame-retardant system. MXene is regarded as a new class of the 2D nanocarbon-based material, which has important theoretical and practical significance for its synergistic effect with halogen-free flame retardants on the flame retardant effect of polymer matrix. Hence, in this work, MXene was prepared and the effect of synergy between MXene and APP on flame retardancy of PVA aerogel was investigated. LOI, UL-94, cone calorimeter tests, and the thermal stability of all samples were investigated. The results showed that the inclusion of MXene can further enhance the flame retardancy of PVA-APP composite aerogels, providing excellent fire and smoke suppression properties.

## 2. Materials and Methods 

### 2.1. Materials

Polyvinyl alcohol (PVA) with a degree of polymerization of 1700 and a saponification degree of 99 mol %, lithium fluoride (LiF), and lithium chloride (LiCl), were purchased from Aladdin Chemicals Co., Ltd. (Shanghai, China). Hydrochloric acid (HCl, 37 wt%) was obtained from Guangzhou Chemical Reagent Factory (Guangzhou, China). Ammonium polyphosphate (APP) was purchased from Clariant Chemicals Co., Ltd. (Guangzhou, China) MAX phase (Ti_3_AlC_2_ 99.7%, 300 mesh) powder was purchased from Nanjing Mission Advanced Materials Co. (Nanjing, China), deionized water was made in the laboratory. All the reagents were used as received.

### 2.2. Synthesis of the Ti_3_C_2_T_x_ (MXene) Nanosheets

The Ti_3_C_2_T_x_ (MXene) nanosheets were synthesized as followed: firstly, 2.0 g of MAX powder was slowly loaded into 40 mL of 3 M LiF and 9 M HCl. Then, the mixture of layered MXene and unetched MAX phase was prepared by continuous stirring at 38 °C for 48 h. The above solutions were washed separately three times with 1 M HCl and LiCl via centrifugation. Subsequently, the solution was centrifuged and washed several times with deionized water until the pH for the supernatant was washed approach to neutral. The precipitates collected by centrifugal washing were distributed in the flask with deionized water and protected with nitrogen for 20 min. The sediment was redispersed with an additional 100 mL of deionized water followed by mixed ultrasonically at ice-bath for 1 h under nitrogen atmosphere. Finally, the solution was then centrifuged for 60 min at 3500 rpm, to give the supernatant slurry containing the exfoliated MXene nanosheets.

### 2.3. Fabrication of PVA-APP-MXene Composite Aerogels

**Scheme 1:**
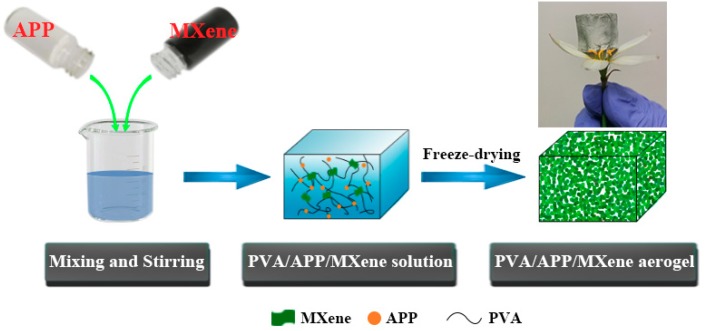
Preparation process for PVA/APP/MXene composite aerogel.

Before processing, PVA and APP were dried in an oven at 80 °C for 24 h. The preparation process for PVA-APP-MXene aerogel was exhibited in Scheme 1. The formulation of PVA composite aerogels was shown in Table 1. A sample containing 85 wt% PVA, 14.5 wt% APP and 0.5 wt% MXene is denoted as PAM-0.5 (where P stands for PVA, A stands for APP and M stands for MXene). In a typical preparation process of PAM-0.5, 85.0 g of PVA powder and 14.5 g of APP were mixed in a certain amount of deionized water and stirred at 85 °C. 0.5 g of net MXene (in the form of colloidal dispersion) were then added to the mixture; it should be noted that the solid content of the mixture was kept at 10 wt%, and mechanically stirred for 4 h at a speed of 200 rpm under a nitrogen atmosphere. Finally, the mixture was poured into the mold to be frozen into ice, following by freeze-dried in a freeze-dryer under a high vacuum (<8 Pa). Before test, all samples were maintained at 80 °C for 24 h under vacuum for desiccation.

### 2.4. Characterization

The microstructure of all samples was determined with a SU8010 scanning electronic microscope (HITACHI, Tokyo, Japan) at 15 kV. The thickness and lateral sizes of MXene were measured with a Multimode Nano 4 atomic force microscope (Bruker, Karlsruh, Germany) in tapping-mode. Intercalated and exfoliated structures of MXene were performed with a D/Max-2500 X-ray diffractometer (Rigaku, Tokyo, Japan) using Cu-Κα radiation (λ = 0.15406 nm). The thermal degradation behavior of all aerogels was executed with a STA449F3 thermogravimeter (NETZSCH, Selb, Germany) from 40 °C to 600 °C with a 10 °C min^−1^ heating rate under 100 mL min^−1^ a nitrogen flow. LOI values were performed with a COI oxygen index meter (Mordis, Kunshan, China) with a dimension of 100 mm × 10 mm × 3 mm according to ASTM D2863-97. The vertical burning test (UL-94) was performed with a CZF-6 instrument (Jiangning, Nanjing, China) using samples with dimensions of 130 mm × 13 mm × 3.2 mm according to ATSM D 3801. Fire behavior was performed with a cone calorimeter device (Vouch, Suzhou, China) with sample dimensions of 100 mm × 100 mm × 3 mm in accordance with ISO 5660 standard processes under a heat flux of 50 kW/m^2^. Raman spectra were recorded with a Confocal Raman Microprobe (Renishaw, London, UK) with a wide scope from 600 to 2000 cm^−1^. The elemental composition of residue was executed with an ESCALAB 250XI spectrometer (Thermo Scientific, Waltham, MA, USA) by using Al-Kα excitation radiation.

## 3. Results and Discussion

### 3.1. Characterization of MXene

**Figure 1:**
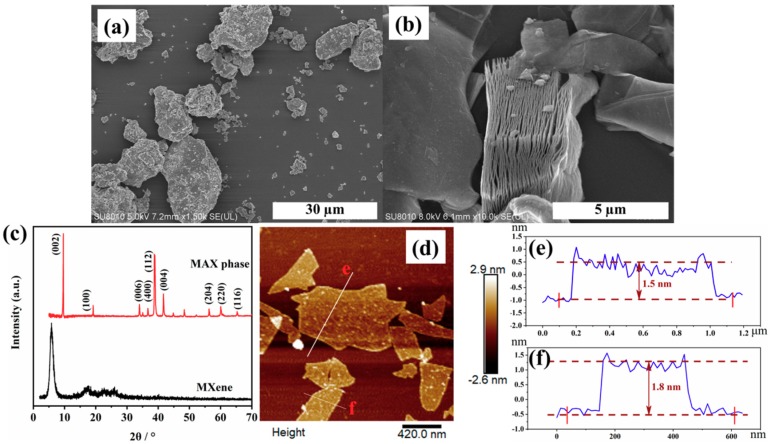
(**a**) SEM image of MAX phase; (**b**) SEM image of non-stripped MXene in low resolution; (**c**) XRD image of MAX phase and MXene; (**d**–**f**) AFM image of stripped MXene and the corresponding height profiles.

Figure 1a shows a SEM image of the MAX phase before etching, which presents tightly stacked flakes and a rough surface. Obviously, after in situ hydrofluoric acid etching, a loosely packed clay-like structure could be observed, which conducive to exfoliating of the MXene sheets owing to the weakened interlayer interactions. It can be known from Figure 1c that the main XRD peak (002) of MAX phase at 2θ = 9.61°, which corresponds to a d-spacing of 9.20 Å. The (002) peak of MXene became broadened and shifted to 5.82° (d = 15.17 Å). The result indicates that the layer spacing between MXene layers can be remarkably increased, due to the intercalation of water molecules and lithium ions (Li^+^) [14]. Moreover, the peak at 2θ = 39° of MXene disappears, further confirming the successful removal Al layers from the MXA phase. The AFM image in Figure 1d exhibits the few-layer structure of the MXene nanosheet with a thickness of 1.5–1.8 nm and lateral sizes up to 600 nm (as shown in Figure 1e,f), which means that it is an ultra-thin 2D material. Hence, the distinctive structure endows the MXene with the capability to uniform dispersed in polymer system.

**Figure 2:**
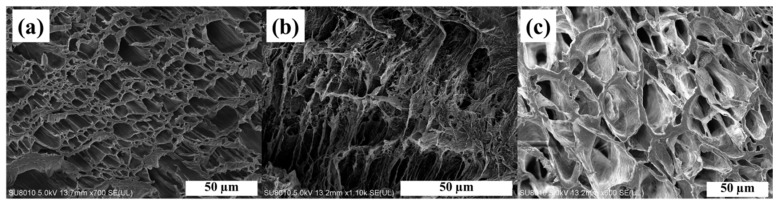
Morphologies of the PVA (**a**), PAM-0 (**b**), PAM-1 (**c**).

The obtained cylindrical PVA composite aerogel can stand upright on the flower without damaging its petals (Scheme 1), indicating that the aerogel is quite light in weight. The density of pure PVA and its composite aerogels has an apparent density between 120.8 and 127.7 mg/cm^3^ (Table 1). Besides, the microstructures of the PVA, PAM-0 and PAM-1 are presented in Figure 2. As shown in Figure 2a, the PVA exhibits a three-dimensional (3D) architecture with an asymmetrical pore distribution. The pore structure is destroyed upon incorporating of APP (Figure 2b), which may be attributed to the poor compatibility of APP fillers with PVA matrix. As can be seen Figure 2c, it can be observed that the addition of MXene results in a more complete 3D pore network structure and a larger specific surface area presumably because of the sustainable effect of the MXene and the strong hydrogen bonding interactions between MXene and PVA [15].

### 3.2. Thermal Degradation Properties

**Figure 3:**
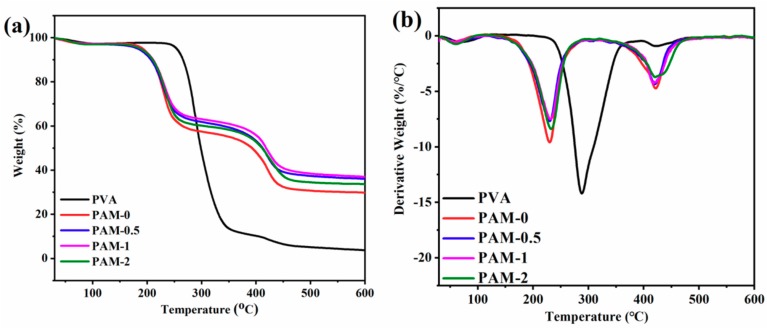
(**a**) TGA and (**b**) DTG thermograms of pure PVA and its composite aerogels in nitrogen atmosphere.

**Figure 4:**
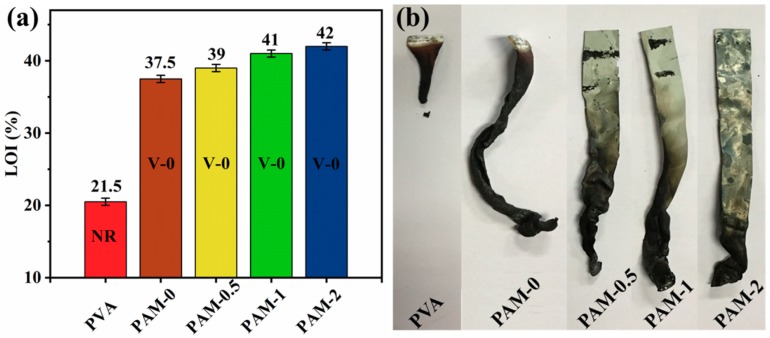
(**a**) LOI, UL-94 test results and (**b**) the photos of the char residues after UL-94 test for pure PVA and its composite aerogels.

The thermal stability of PVA and its composite aerogels were investigated by TGA under nitrogen. Figure 3 displays thermogravimetric tests results, and the relevant data are summarized in Table 2. Three-stage degradation processes for pure PVA and its composite aerogels are observed in Figure 4b. The first stage from below 100 °C is assigned to the degradation of release of water from the aerogel [16]. The main mass loss stage, between 180 and 400 °C, is ascribed to the degradation of the main chains of PVA. The final mass loss stage is range from 400 to 480 °C, which is ascribed to the further pyrogenic decomposition of the unstable residues created in the main stage. The PVA including APP or a combination of MXene and APP exhibit lower initial degradation temperatures (T_5%_) and maximum degradation temperatures (T_max-1_) than the pure aerogel. This may be ascribed to two reasons: on the one hand, the existence of APP promotes the thermal decomposition of the aerogels [17]; on the other hand, the decomposition of MXene surface termination groups [13]. The sample containing MXene show a higher residue at 600 °C than the PAM-0 sample, which implying the introduction of MXene promotes charring and partial polymer cannot be completely burned, leading to enhanced the char residues. Furthermore, in Figure 3b and Table 2, the maximum mass loss rates of PAM-1 composite aerogel is much lower than that of PAM-0. This indicates that MXene inhibits the decomposition of the polymer and promotes the rapid carbon formation of the PVA-APP system, suppressing the diffusion of heat and fuel, thereby enhancing the flame-retardancy of the aerogels.

### 3.3. Fire Behavior

The LOI and UL-94 tests of the PVA composite aerogels were conducted, and the corresponding results are displayed in Table 1. PVA aerogel is quickly combustible for its LOI value only 20.5%, and failed the UL-94 test. The inclusion of 15 wt% APP to PVA aerogel result in a strongly improve LOI value (+17%). In the flame retardant PVA systems, partial substitution of APP by MXene leads to further improves in LOI. The LOI values are 39, 41, and 42%, corresponding to the samples with 0.5, 1, and 2 wt% MXene content, respectively. Figure 4 shows digital photographs of the residues of pure PVA and the composite aerogels after UL-94 test. 

As observed, the pure PVA is a flammable polymer with serious dripping. All samples with added APP/MXene reached a V-0 rating in the UL-94 test. It can be observed that intumescent residues are formed at 15 wt% APP loading. Furthermore, with the incorporation of MXene, the degree of combustion of the sample during vertical combustion is reduced and the residue is more compact compared with PAM-0. Therefore, MXene can effectively enhance charring of the PVA-APP system. This phenomenon is also in accordance with the results of the previous TGA test.

**Figure 5:**
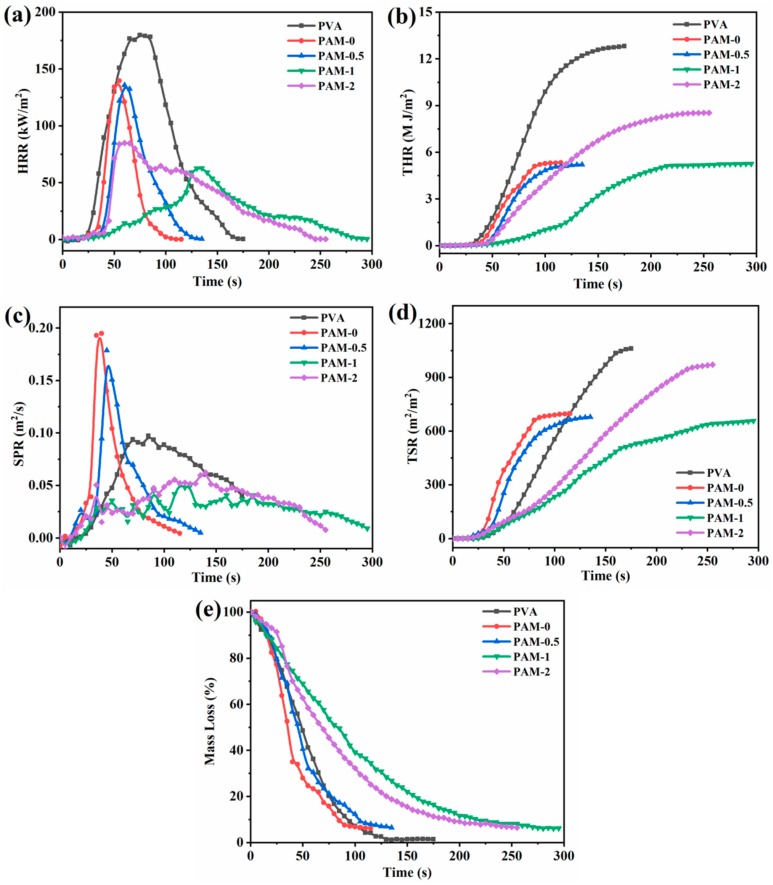
HRR (**a**), THR (**b**), SPR (**c**), TSP (**d**), and mass loss (**e**) as a function of the burning time for PVA and its composite aerogels.

The fire behaviors of PVA and its composites are estimated by the cone calorimeter. Important fire-hazard parameters containing time to ignition (TTI), time to peak heat release rate (t_pHRR_), the peak heat release rate (pHRR), total heat release (THR), peak smoke production rate (PSPR), total smoke production (TSP), the maximum average rate of heat emission (MARHE), the effective heat of combustion (EHC), and mass loss are tested. Figure 5 shows HRR, THR, PSPR, TSP, and the mass loss curves of all samples at a heat flux of 50 kW/m^2^ and Table 3 summarizes the related data.

Figure 5a,b shows the HRR and THR curves of all samples, respectively. Pure PVA aerogel is rapidly burned after ignition, with highest values of pHRR and THR of 180 kW/m^2^ and 12.8 MJ/m^2^, respectively. Compared with pure PVA aerogel, the pHRR values of the APP-containing composite aerogel decreases to 140 kW/m^2^. Moreover, the pHRR values is further declined when the APP is replaced by MXene. The pHRR values of PAM-0.5, PAM-1, PAM-2 are reduced by 3.6%, 55%, 39.3%, respectively, in comparison with PAM-0 sample. Notably, the HRR curves exhibit a flat progress when the loading amount of MXene exceeds 0.5 wt%, illustrating that the aerogels with APP and MXene burnt more slowly. This suggests that MXene could reduce the HRR of PVA-APP system. However, further increasing the MXene loading was unfavorable to the reduction in the PHRR. The results demonstrate that only at the optimum loading MXene can achieve the flame retardant synergistic enhancement effect. The lower or higher loading of MXene has an antagonism effect on the APP and the THR value of PAM-0.5 is further reduced to 5.2 MJ/m^2^, which suggests that part of PVA matrix is incompletely burned. Furthermore, the incorporation of APP or MXene can delay the t_pHRR_. Especially, the t_pHRR_ of the sample with introduction of 14.0 wt% APP and 1.0 wt% MXene reached 135 s, which is greatly prolonged by 60 s compared with the t_pHRR_ of pure PVA, which can increase the escape time and the probability of escape in case of fire.

SPR and TSR curves of all samples are displayed in Figure 5c,d, respectively. The TSR value of PAM-0 is drastically reduced from 1035 m^2^/m^2^ for pure PVA to 682 m^2^/m^2^, which corresponds to a 34% reduction. However, the PSPR value of PAM-0 is higher than that of pure PVA, which may be due to the decomposition of APP that can release massive amounts of NH_3_ and other gases during the burning procedure [18]. Compared with pure PVA, the PSPR and TSP of PAM-1 decreased by 50% and 38%, respectively. The results confirm that MXene can effective enhance smoke production suppression during burning of PVA-APP systems. Hence, the likelihood of suffocation is reduced during the fire escape.

Figure 5e shows the curves of mass loss versus burning time, and the residue yield is shown in Table 3. The mass of pure PVA decreased rapidly, suggesting that the sample is burned completely. while the PAM-0 sample shows a faster decline in mass loss, indicating that APP can promote char formation, but 15 wt% APP is not enough to hinder the combustion of PVA. At the same time, the burning rate is reduced with the introduction of different contents of MXene, of which PAM-1 has the slowest burning rate, indicating that 1.0 wt% MXene can best delay the burning of the system. This is accordance with the heat and smoke release results.

The maximum average rate of heat emission (MARHE) and the fire performance index (FPI) are used to evaluate the fire hazards of aerogels [19,20]. In general, higher FPI and lower MARHE values mean superior fire safety of polymers. The FPI values of PVA, PAM-0, PAM-0.5, PAM-1, PAM-2 were 0.07, 0.16, 0.19, 0.22, 0.59, respectively. It can be observed that the loading of MXene significantly enhanced the FPI value of the system. The MARHE value of PAM-1 is 32.4 kW/m^2^, which is the lowest among all samples. Both FPI and MARHE results suggest that the combination of MXene and APP can be highly efficient to improve the fire safety of PVA.

To highlight the significant effect in fire safety and smoke suppression performance of polymers by MXene, a comparative performance evaluation of PVA composite aerogel results of reported MXene-based polymers and PVA containing other flame retardants was performed, as shown in Table 4. Obviously, although the addition of a single MXene or modified MXene can reduce the PHRR of the system, the THR is reduced slightly [13,21]. MXene/chitosan nanocoating is conductive to suppressing the release of heat and smoke in TPU via the layer-by-layer (LbL) approach, but there is drawback in the complex manipulation [22]. Moreover, traditional nanomaterials can effectively improve the flame retardant performances of the system, however, there they have defects such as large addition or the need for surface modification of the nanomaterials [23,24,25,26,27]. In this work, with our simple strategy without any chemical modification, the introduction of MXene and APP to PVA leads to a significant decrease in PHRR (55%), THR (33.6%) and PSPR (74%), which are better than majority of results reported in the literature, suggesting the superior flame retardant and smoke suppression performance of the proposed combination.

### 3.4. Analysis of the Residue

**Figure 6:**
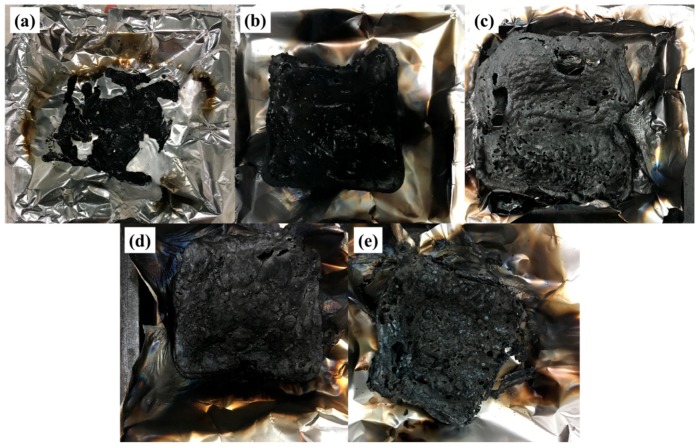
Photographs of residues of (**a**) PVA (**b**) PAM-0, (**c**) PAM-0.5, (**d**) PAM-1, and (**e**) PAM-2 at flame-out after burning.

**Figure 7:**
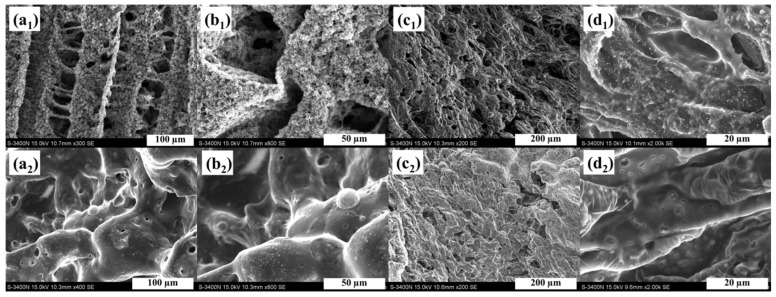
SEM images of the (**1**) outer and (**2**) inner of char residues for PAM-0 (**a**,**b**) and PAM-1 (**c**,**d**).

Figure 6 exhibits digital photographs of the residues for all aerogels after the cone calorimeter tests. As shown in Figure 6a little residues are left in the pure PVA sample. The residues of PAM-0.5 and PAM-1 are significantly higher than that of PAM-0, indicating that a low addition of MXene enhances the formation of intumescent residue. Moreover, it can be observed that the PAM-1 sample exhibits a more compact and continuous residue, leading to more efficient protection. To further understand the morphology of the residue microstructure, the morphology of the outer (Figure 7a_1_–d_1_) and inner (Figure 7a_2_–b_2_) residues for PAM-0 and PAM-1 were examined using SEM under different magnifications. A continuous char structure with obvious holes and cracks is observed in the outer surface of PAM-0 char (Figure 7a). As compared to the PAM-1 sample, where the outer residue exhibits a compact and complete surface with compressed layer (Figure 7). The SEM images of inner residues (Figure 7a_2_–b_2_) further confirm that the inner char residues of PAM-1 is more compact than that of PAM-0. The char residues of PAM-1 display a more cohesive and pleated layer on the surface with no holes and cracks. As is known to all, a cohesive and continuous surface of residues is conductive to inhibiting the diffusion of heat and inflammable gases, thus enhancing the fire safety [31].

**Figure 8:**
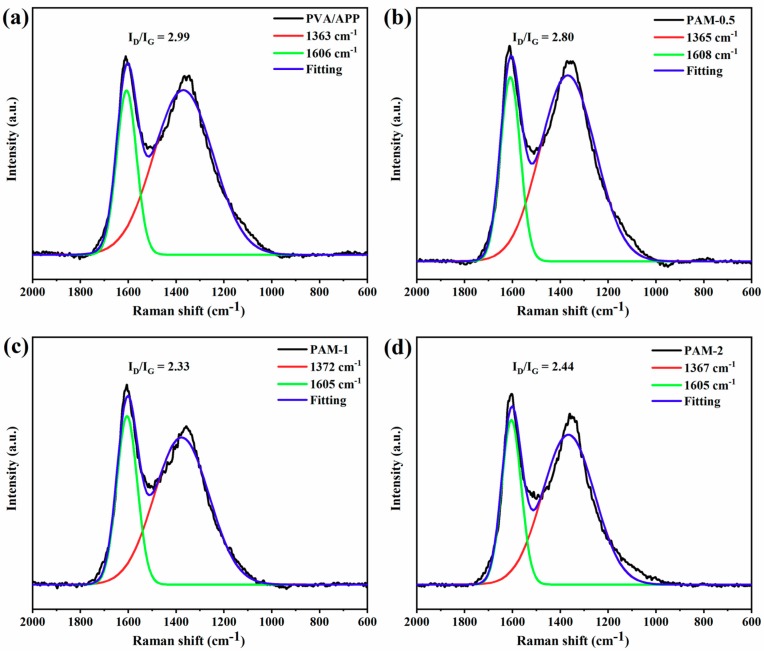
Raman spectra of the char residues of PVA composite aerogels (**a**) PAM-0, (**b**) PAM-0.5, (**c**) PAM-1, and (**d**) PAM-2.

Raman spectroscopy was performed to investigate the degree of graphitization of the residues after cone calorimetry. Figure 8a–d present the Raman spectra of the residues of the PVA composite aerogels. The degree of graphitization of the residue can be investigated by the intensity ratio of the D (peaks at approximately 1360 cm^−1^) and G (peaks at approximately 1590 cm^−1^) bands (I_D_/I_G_). The carbonaceous material with higher graphitization degree indicates higher thermal oxidation stability, which is conductive to enhancing the flame retardancy of polymers. Generally, a higher graphitization degree corresponds to a lower I_D_/I_G_ value. The incorporation of MXene increases the I_D_/I_G_ value of the PVA-APP system, indicating an increase in the degree of graphitization in the residues.

Moreover, the I_D_/I_G_ of PAM-1 is higher than those of PAM-0.5 and PAM-2, suggesting that the residues for PAM-1 as a barrier can have a higher efficiency in preventing heat and fuel diffusion between the condensed phase and the gas phase, thus inhibiting the burning of matrix, and reduce the HRR during burning.

**Figure 9:**
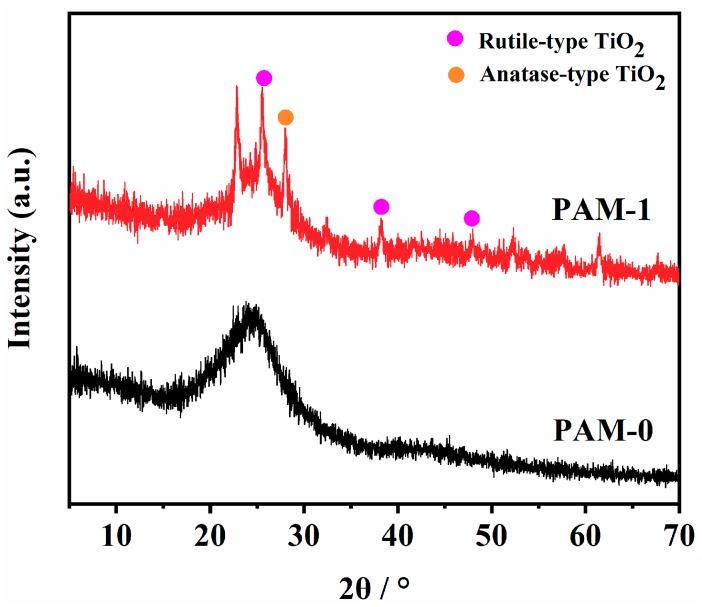
XRD patterns of the char residues of PAM-0 and PAM-1 composite aerogels.

XRD is used to detected the phase composition of the char residues after burning. As shown in Figure 9, it can be observed that a broad diffraction peak at 24.6° is present for the PAM-0, which is ascribed to the crystalline diffraction peak of graphite. 

Due to the existence of MXene in the char residue of PAM-1, several new peaks appeared compared to PAM-0. The peaks labelled with purple dot (25.5°, 38.3°, 48.0°) and orange dot (28°) are ascribed to anatase-type and rutile-type TiO_2_, respectively [32]. This phenomenon demonstrates that Ti-containing of MXene undergoes thermal oxidation to form stable TiO_2_ during burning. Moreover, the TiO_2_ may enhance the charring and cross-linking of APP and PVA by catalysis, which further improvez the formation of compact residual char [33].

**Figure 10:**
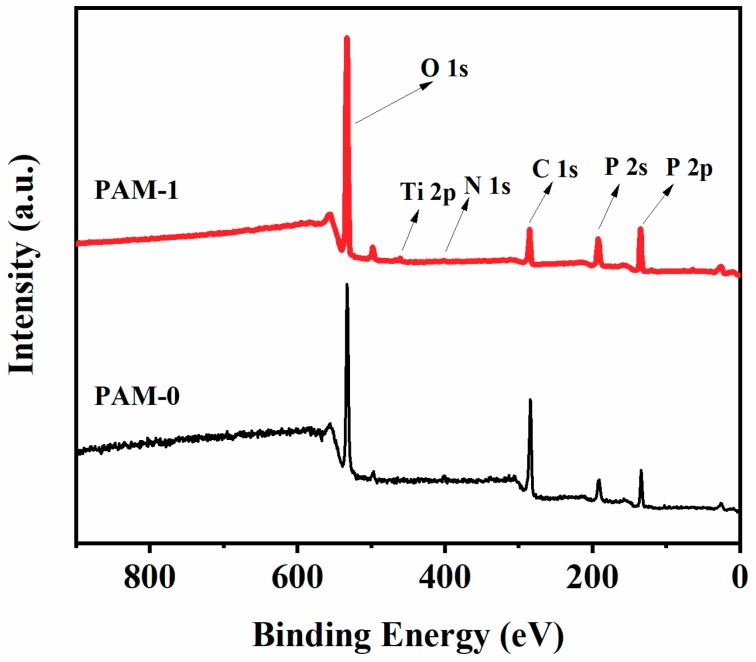
Overall XPS spectra of the char residues after burning in cone calorimeter tests.

To further analyze the synergistic mechanism of MXene and APP, XPS was applied to determine the elemental composition and content of PAM-0 and PAM-1. The overall XPS spectra for the residues of the PAM-0 and PAM-1 are shown in Figure 10, and the elemental composition is presented in Table 5. The P/C and N/C ratios of PAM-1 are higher than PAM-0, suggesting an enriched N and P-containing on the residues of PAM-1, which further indicate that the barrier effect of MXene may restrain the release of NH_3_ and decrease the volatility of P, and thus more N and P elements are retained in the residues, which is conductive to the formation of more continuous and cohesive residues [22].

**Figure 11:**
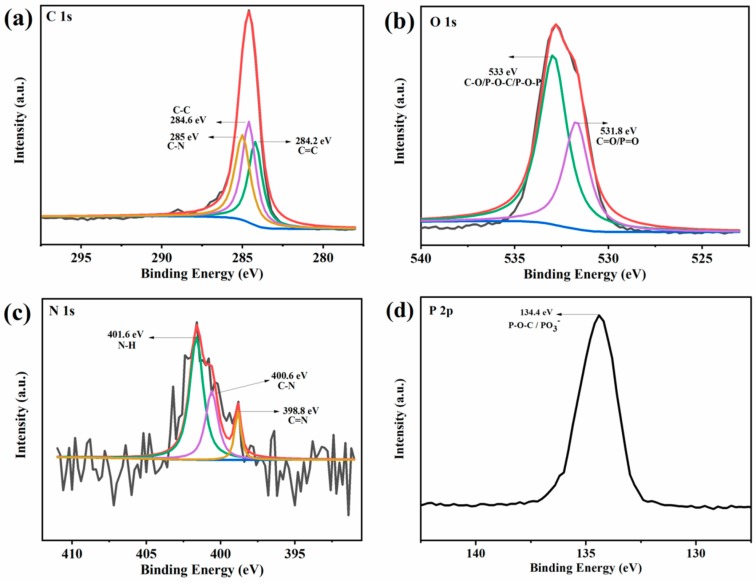
(**a**) C 1s, (**b**) O 1s, (**c**) N 1s, and (**d**) P 2p XPS spectra of the residues for PAM-0.

**Figure 12:**
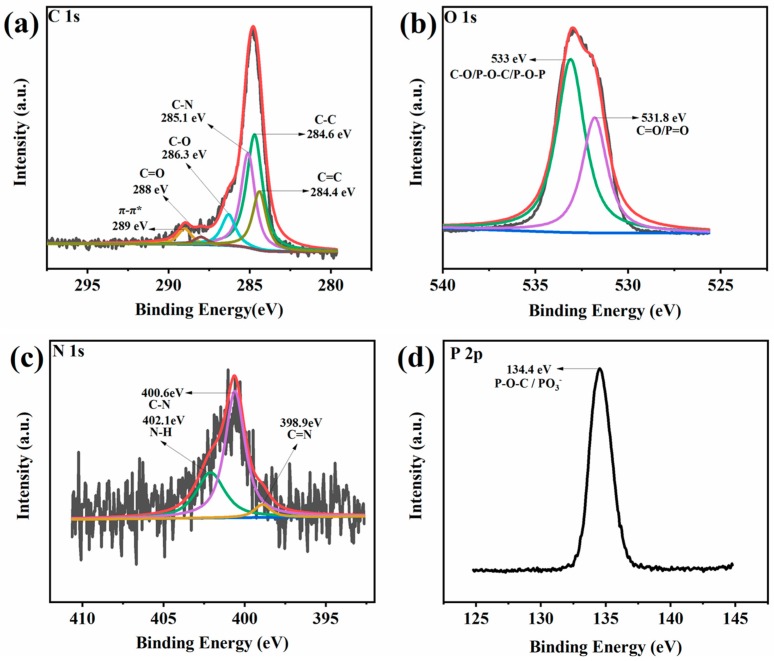

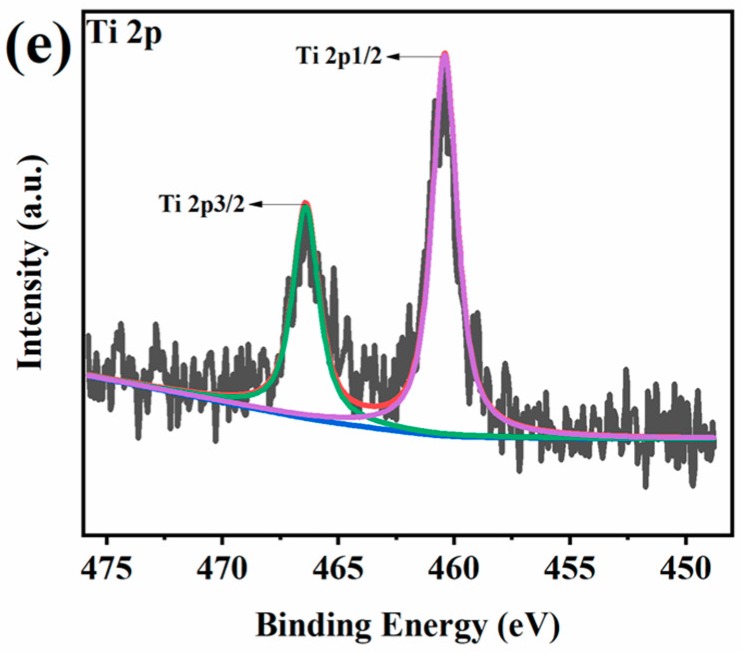
(**a**) C 1s, (**b**) O 1s, (**c**) N 1s, (**d**) P 2p, and (**e**) Ti 2p XPS spectra of the residues for PAM-1.

High-resolution XPS spectra for PAM-0 and PAM-1 are presented in Figure 11 and Figure 12. As presented in the C 1s spectrum of PAM-0, the peaks appearing at 284.2, 284.6 and 285 eV correspond to C=C, C–C and C–N bonds. As presented in the O 1s spectrum, the two characteristic peaks at 531.8 and 533 eV are due to C=O/P=O and C–O/P–O–C/P–O–P, implying the formation of crosslinking P-containing structures [31]. The N 1s spectrum is divided into three peaks including C=N, C–N and N–H at binding energies of 398.8, 400.6 and 401.6 eV [34,35], indicating the charring reaction of PVA and APP. In the P 2p spectrum, the signal peak at 134.4 eV is due to P–O–C/PO_3_^-^ in phosphorus-rich crosslinks [36], while for PAM-1, there are three new peaks at 286.3, 288, 289 eV in its C 1s spectrum, which are attributed to C–O, C=O and π–π*, respectively. This suggests that MXene inhibits the decomposition and oxidation of PVA, thereby reducing the release of volatilized gases, such as CO, CO_2_, carbonyl compounds, etc [37]. Moreover, the additional element (Ti 2p) signal is split into two peaks: 460 eV (Ti 2p1/2) and 466.5 eV (Ti 2p3/2), which reveals the presence of Ti-containing substances. These suggest that the MXene in PVA-APP system can reduce polymer degradation and the release of volatile gases, thus enhancing the fire safety.

### 3.5. Flame Retardant Mechanism

**Scheme 2:**
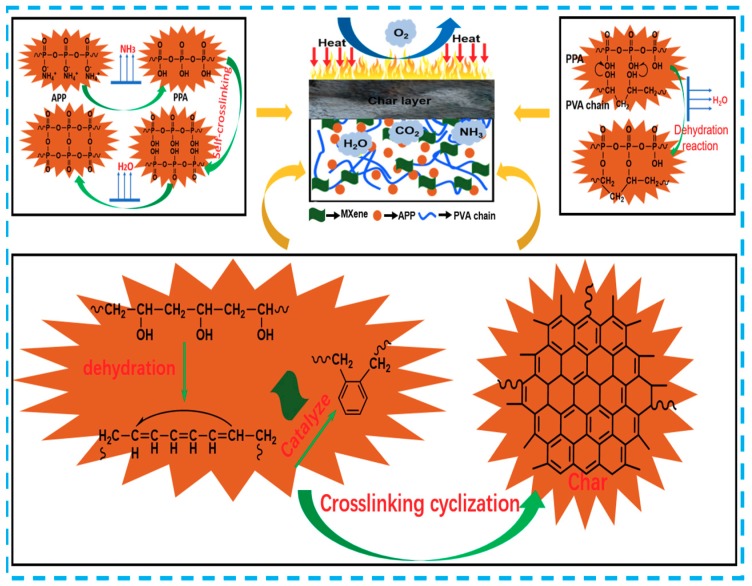
Schematic illustration for the synergistic flame-retardant mechanism of PVA-APP-MXene composite aerogels.

Based on all the above analysis, a possible mechanism of the synergistic interaction between MXene and APP in PVA aerogels is shown in Scheme 2. When the aerogels are ignited, APP begins to decompose from PPA with the release of ammonia (NH_3_) and water (H_2_O), thus diluting the inflammable gases in the combustion system and formation of intumescent residues, which will decrease the heat release and delay burning [38]. At the same time, a self-crosslinking reaction occurred in part of the PPA. Moreover, PPA may be dehydrated on the PVA chain, which promotes the formation of a stable residue [39]. In this work, MXene would be oxidized on its surfaces and/or edges and generate ultra-small anatase TiO_2_ particles due to the strong tendency of the Ti component to react with oxygen during burning, thus consuming oxygen and inhibiting the burning of the matrix. TiO_2_ may further boost the cross-linking and charring of APP and/or PVA and promote the formation of compact intumescent residues via catalytic effects [13]. Furthermore, the MXene and APP strengthened the polymer melt viscosity of PVA and enhanced the formation of a compact Ti, P and N hybrid residual char in burning, which as a barrier that inhibits the aerogel from further combustion and restrains the release of smoke and heat [22].

## 4. Conclusions

Herein, PVA/APP/MXene composite aerogels were fabricated by a freeze-drying approach. The feasibility of employing MXene as a synergist for APP was investigated in the flame retardancy aspect. With the incorporation of MXene, the fire performance of the PVA aerogels is significantly improved, and adding 1.0 wt% MXene into PVA-APP system leads to a distinct enhancement of the residue yield and a remarkable decrease of the maximum thermal decomposition rate; moreover, MXene can also act as an effective agent for decreasing heat release and suppressing smoke release. In addition, MXene could enhance the degree of graphitization and promote the formation of a continuous char structure. A synergistic flame-retardant mechanism was proposed based on the analyses of the condensed phase residues produced by burning. Furthermore, due to the presence of the MXene, which catalyzes the formation of char, the Ti, P and N hybrid residual char prevents the transform of the heat and fuel, thus improving the flame retardancy of the polymer materials.

## Figures and Tables

**Table 1:** Formulation and apparent density of pure PVA and its composite aerogels.

Samples	PVA (wt%)	APP (wt%)	MXene (wt%)	Apparent Density ^1^ (mg/cm^3^)
PVA	100	-	-	124.4 ± 0.9
PAM-0	85	15	-	127.7 ± 0.8
PAM-0.5	85	14.5	0.5	126.0 ± 1.1
PAM-1	85	14.0	1.0	121.3 ± 0.7
PAM-2	85	13.0	2.0	120.8 ± 0.8

^1^ Apparent density was calculated from the quality and volume of the aerogel.

**Table 2:** TGA results for all samples in nitrogen.

Sample	T_5%_ (°C)	T_max-1_ (°C)	T_max-2_ (°C)	Residue at 600 °C (wt%)
PVA	254	288	424	3.7
PAM-0	192	230	422	29.8
PAM-0.5	183	230	420	36.0
PAM-1	189	232	417	37.0
PAM-2	188	233	421	33.6

**Table 3:** Cone calorimeter results of the PVA and its composite aerogels.

Sample	TTI (s)	t_pHRR_ (s)	pHRR (kW/m^2^)	THR (MJ/m^2^)	MARHE (kW/m^2^)	Mean-EHC (MJ/kg)	^a^ FPI ((m^2^·s)/kW)	PSPR (kW/(m^2^·s))	TSR (m^2^)	Residue (wt%)
PVA	13	75	180	12.8	99.4	8.96	0.07	0.10	1035	1.4
PAM-0	22	55	140	5.3	50.7	9.55	0.16	0.19	682	6.22
PAM-0.5	26	60	135	5.2	49.2	9.58	0.19	0.18	655	6.5
PAM-1	14	135	63	5.3	32.4	3.87	0.22	0.05	646	6.2
PAM-2	50	60	85	8.5	45.1	9.35	0.59	0.06	945	6.5

^a^ FPI = TTI/pHRR.

**Table 4:** PHRR, THR, PSPR, LOI and UL-94 results of MXene based polymer and PVA composites contained other flame retardant in previous work and this work.

Matrix	Flame Retardant	Content (wt%)	PHRR Reduction (%)	THR Reduction (%)	PSPR Reduction (%)	LOI (%)	UL-94	Ref.
TPU	TBPC-Ti_3_C_2_	2	52.2	1.5	57.4	-	-	[13]
PS	OTAB-Ti_3_C_2_	2	26.4	-0.09	-	-	-	[21]
PVA	MXene	1	2.6	25.5	-	-	-	[28]
PUF	MXene/Chitosan	-	57.2	65.5	60.3	-	V-0	[22]
PVA	ZrP-EA	8	42.1	16.7	-	-	-	[29]
PVA	HAC	20	-	-	-	30.7	V-0	[30]
PVA	APP/MMT	14.3/0.7	72.7	56.6	-	30.8	V-0	[25]
PVA	MMT/Gelatin	41.7/16.7	13.0	0.68	-	28	-	[26]
PVA	Clay/APP	41.7/16.7	36.8	-36.7	-	-	-	[27]
PVA	APP/MXene	14/1	55	33.6	74	41.0	V-0	This work

TPU: thermoplastic polyurethane; PS: Polystyrene; PUF: Polyurethane foam; TBPC-Ti_3_C_2_ and OTAB-Ti_3_C_2_: Inorganic modification Ti_3_C_2_; ZrP-EA, HAC: Organically modified α-ZrP; MMT: Montmorillonite.

**Table 5:** XPS data of the char residues of PAM-0 and PAM-1.

Sample	C (at%)	O (at%)	N (at%)	P (at%)	Ti (at%)	P/C	N/C
PAM-0	43.90	44.60	2.30	9.20	0	0.21	0.052
PAM-1	19.48	62.44	1.41	15.95	0.72	0.82	0.072
